# The transition of eldercare responsibility and traditional filial piety concepts and its urban-rural differences in China: an age-period-cohort analysis from 2006 to 2017

**DOI:** 10.1186/s12889-024-19175-5

**Published:** 2024-06-22

**Authors:** Xiangxiang Liu, Hong-jie Yu, Min-zhe Zhang, Hong-guang Yang, Rui Chen, Litao Zhao, Qi-qiang He

**Affiliations:** 1 National Clinical Research Center for Infectious Diseases, Shenzhen Third People’ s Hospital, Shenzhen, China; 2 School of Public Health, Wuhan University, Wuhan, China; 3 East Asian Institute, National University of Singapore, Singapore, Singapore; 4 Hubei Biomass-Resource Chemistry and Environmental Biotechnology Key Laboratory, Wuhan University, Wuhan, China

**Keywords:** Eldercare responsibility, Filial piety, Old-age pension, Age-period-cohort analysis

## Abstract

**Background:**

With rapid urbanization, massive migration, and non-family–based eldercare involvement, Chinese concepts of eldercare responsibility and filial piety are shifting. We performed age-period-cohort (APC) analyses to assess the transition of old-age pension coverage, eldercare responsibility, and filial piety concepts and its urban-rural differences among Chinese adults using data from the China General Social Survey (2006–2017).

**Methods:**

Old-age pension coverage (yes/no) and primary eldercare responsibility (government/offspring/self/sharing) were investigated in 2010, 2012, 2013, 2015, and 2017. Filial piety was evaluated using customized questionnaires in 2006 and 2017. The APC effects were estimated using mixed effects and generalized additive models.

**Results:**

Among 66,182 eligible participants (mean age: 48.8 years, females: 51.7%) in the six waves, APC analyses indicated that old-age pension coverage increased with aging and over time. Across cohort groups, it grew as the cohort was younger in urban residents but decreased in rural residents. The concept of offspring-based (> 50%) and government/self/offspring-shared eldercare (> 30%) predominated. APC analyses revealed that the offspring-based concept declined with aging (OR = 0.81, 95% CI: 0.79–0.84), whereas the government-based (OR = 1.37, 95% CI: 1.33–1.41) and self-based (OR = 1.55, 95% CI: 1.47–1.63) concepts increased with aging. People born around the 1940s have a comparatively higher possibility to perceive that the primary eldercare responsibility should be undertaken by the government and elder parents. In contrast, people born in the younger cohort were more likely to perceive that adult children are responsible for their parents’ primary eldercare. Filial piety score slightly increased with aging (β = 0.18, SD: 0.05) but decreased as the birth cohort was younger. In addition, rural participants were more likely to perceive offspring-based eldercare and maintain filial piety, and the related urban-rural difference was intensified by aging.

**Conclusions:**

The traditional concept that eldercare solely relies on offspring has changed to relying on multiple entities, including the government and self-reliance. Diluted filial piety in people born in the young cohort requires reinforcement. Moreover, future healthy aging policies need to focus more on urban-rural disparities to promote equity in social well-being.

**Supplementary Information:**

The online version contains supplementary material available at 10.1186/s12889-024-19175-5.

## Background

Offspring-based eldercare was an inherent social norm during the thousands of years of Chinese civilization, and it is widely eulogized as “filial piety” in Chinese *Confucian* ethics [1]. Filial piety briefly refers to unconditional economic and emotional support for parents [1]. The concept that “raise children to fight against aging” and “offspring undertake the primary eldercare responsibility” has been rooted in traditional culture for a long time [1, 2]. This intergenerational contract was stipulated as a legal obligation since China’s new marriage law was promulgated in 1950 [1]. However, a range of social changes, including migration, urbanization, and old-age pension implementation, gradually influenced the Chinese attitude toward eldercare responsibilities and patterns and traditional filial piety concepts.

Massive migration related to economic development and urbanization has gradually shaken the leading role of offspring-based eldercare and eroded traditional filial piety. The population of migrant workers has steadily increased in recent decades, reaching 172 million in 2022 in China [3]. It was reported that approximately half of the elderly population (118 million) in 2020 [4, 5] and 48.6% of children (67 million) in 2022 [3] in China were left behind. Labor migration hinders the healthy aging of empty nest elderly because filial piety behaviors are difficult to implement, particularly for the physical care and emotional support to parents [5]. For left-behind children, neglectful parenting styles caused by migration are unfavorable to perceive the interdependence among family members and cultivate filial beliefs [6]. Currently, younger laborers with high education attainment also migrate continuously from villages or small cities to megacities [7, 8]. Coastal provinces and municipalities absorbed over 77% of migrants nationwide [7]. On the one hand, expensive living and child-rearing costs for migrants living in megacities result in an unreasonable financial burden to fail to provide extra economic support for their parents or even exhaust parental properties to survive in urban life [9]. On the other hand, the traditional eldercare expectation of elder parents relying on children altered to self-reliance and the avoidance of becoming the burden for offspring [10]. China experienced the most significant rural-to-urban migration worldwide from 1978 to 1999 (the era of Reform and opening-up in China) [11]. An empirical study demonstrated that more advanced city modernization and higher education levels of citizens lead to a lower level of filial piety, particularly among those who grew up in this era [12]. 

Furthermore, the concept of eldercare responsibility and filial piety has been shifted within the implementation of continuous social policies, including the development and reform of the old-age pension scheme and the One-Child policy. The Chinese modern old-age pension scheme has gradually popularized since 1997 and covered half of the urban employees (roughly accounting for 30% of total urban residents) by 2005 [13]. The “New Rural Pension Scheme (NRPS)” for the rural population and the “Urban Resident Pension Scheme (URPS)” for non-employed urban residents were issued in 2009 and 2011, respectively [13]. They were combined as a unified old-age pension scheme in 2014 and have almost achieved universal coverage [14]. Benefiting from that and the increased personal wealth, self-reliance in eldercare becomes more achievable regarding substance requirements [1, 15]. In addition, the One-Child Policy between 1980 and 2015 embedded with migration and urbanization extensively shrank the family size with a substantial increase in the one-couple and two-generation nuclear households [16, 17]. Consequently, multiple eldercare patterns, including community and institutional eldercare, are booming [1, 15, 18]. In China, the number of older adults with institutional eldercare is projected to increase from 200 million in 2015 to 290 million in 2035 [19]. Furthermore, the stigmatization of placing parents in institutions for eldercare has been replaced with independent and autonomic living arrangements in both young and old adults [1, 18]. However, under these social policies, how Chinese filial piety changed remained controversial. Several studies have indicated that filial piety weakened during the transformation of the economy and politics [12, 20, 21], and the concept that the government should bear more eldercare responsibility has strengthened [22]. In contrast, a few studies demonstrated that filial piety was reinforced and active in adapting to modernized social life, particularly in the younger birth cohort [23, 24]. It is also probable that filial piety remained unaffected because it is rooted in Chinese culture [1, 15]. Clarifying the transition of the filial piety concept and its intergenerational difference is imperative to better serve long-term eldercare for the rapid aging of the Chinese population.

Taken together, these social changes, including migration, urbanization, and social policy implementation, may limit filial practices and challenge the dominant role of the offspring-based eldercare pattern [25]. Moreover, people born/raised within these social changes may develop or reshape eldercare perceptions and filial piety [1, 12]. Studies have also observed age-specific preferences for eldercare patterns, with older elderly people favoring institutional care compared with young elderly people [26, 27]. The Chinese elderly population (65 years or older) reached 172 million and accounted for 12% of the total population in 2020, which is projected to increase to 366 million (26.1%) by 2050 [28, 29]. Facing such a rapidly aging society, the assessment of the difference in the concept of primary eldercare responsibility and filial piety across different birth cohorts and age groups is needed, which will benefit the understanding of the dynamic impact of those social changes on eldercare patterns and concepts, as well as prompt the well-being of older adults and social harmony [15, 17, 18, 29, 30]. We hypothesized that (i) young adults and those who grew up after the 1980s were more likely to perceive that eldercare responsibility should be primarily shared by the government, adult children, and older parents; and (ii) they have a lower level of filial piety.

This study aimed to characterize the time trends and intergenerational differences in old-age pension coverage, primary eldercare responsibility, and filial piety concepts through age-period-cohort (APC) models using the Chinese General Social Survey (CGSS) from 2006 to 2017. Given that filial piety and related perceptions evolved distinctly between urban and rural areas [1, 15, 20], all APC analyses considered urban-rural differences.

## Materials and methods

### Data source and participants

The CGSS is a nationally representative survey that has been repeated annually or biannually since 2003 to investigate the association between multidimensional social issues and quality of life in urban and rural China [31]. The CGSS recruited adults (aged 18 years and above) using a multistage stratified sampling method [31]. The details of the study design and research ethics are shown on its survey website (http://cgss.ruc.edu.cn/English/Home.htm). Information on the old-age pension scheme and primary eldercare responsibility was available in 2010, 2012, 2013, 2015, and 2017; the filial piety score was only available in 2006 and 2017. The current study selected adults aged 20–90 years who had complete information on outcome variables, age, and covariates (including sex, ethnicity, registered residence, education, and marital status). Participants who answered questions by proxy were excluded.

### Dependent variables

Old-age pension scheme coverage (yes/no) was assessed by asking participants whether they participated in the rural or urban old-age pension scheme.

Primary eldercare responsibility and filial piety in the CGSS have been widely used in the analysis of multiple family issues, including intergenerational relationships [32, 33] and subject well-being [34] of family members. Primary eldercare responsibility was investigated by asking the participants’ opinion on “Who should undertake the primary eldercare responsibility?” with four responses: (i) government, (ii) offspring, (iii) themselves, and (iv) sharing among them [15]. Correspondingly, we coded it as four dummy variables.

Filial piety was assessed by asking participants to rate their responses (seven-point Likert-scale from “strongly disagree” to “strongly agree”) to the seven following descriptions [24]: (i) Offspring should respect father’s authority in any case; (ii) Offspring should give birth to at least one male heir to preserve the family lineage; (iii) Offspring should do something that makes parents feel proud; (iv) Offspring should be grateful to parents for upbringing; (v) Offspring should treat parents well no matter how they behave; (vi) Parents’ opinion is superior to offspring’s aspiration; (vii) Supporting parents to make their senior living more comfortable. The sum score ranges from 7 to 49, with a higher score indicating more traditional filial piety.

### Independent variables

Age was calculated as the study period minus the birth year and was included as a continuous variable. The study period was included as an ordinal categorical variable. The birth year was grouped into 12 birth cohorts and also involved as an ordinal categorical variable: before 1939, 1940-4, 1945-9, 1950-4, 1955-9, 1960-4, 1965-9, 1970-4, 1975-9, 1980-4, 1985-9, and after 1990. The birth year was derived from the census record [31]. 

### Covariates

Sex (male, female), ethnicity (Han, minorities), registered residence (urban, rural), education (primary school and lower, middle school, high school, and university and higher), and marital status (unmarried, married/partnership, divorced/widowed) were investigated by face-to-face interviews.

### Statistical analysis

Descriptive statistics are presented as mean ± standard deviation for age and traditional filial piety score, and frequency (%) for all covariates and the concept of old-age pension coverage and primary eldercare responsibility. All analyses were performed using *R* software 4.2.1.

### APC based on mixed effects and generalized additive models

The APC model has been widely used to analyze repeated cross-sectional and long-term panel data [35]. Age (A) effects refer to the difference in potential outcomes caused by the aging process specific to individuals; Period effects (P) represent the difference resulting from the periods of observation or measurement; and cohort effects (C) indicate the difference linked to the year of birth or common experience specific to years [36]. The linear dependency among these three components because of “Period–Age = Cohort” limited its reliability and was termed an “identification problem.” [36] The mixed effects model assigns period and cohort as random effects and age as a fixed effect to handle cross-level interactions [36], known as the hierarchical APC (HAPC) model. Referring to the HAPC framework [36], we designed a two-level model specified as follows:

Within-cell model (Level 1)

1 $$\begin{gathered} Dependent\,variable{s_{ijk}} = {\beta _0}jk + {\beta _1}Ag{e_{ijk}} + {\beta _2}Ag{e^2}_{ijk} + \hfill \\\,\,\,\,\,\,\,\,\,\,\,\,\,\,\,\,\,\,\,\,\,\,\,\,\,\,\,\,\,\,\,\,\,\,\,\,\,\,\,\,\,\,\,\,\,{\beta _n}Co\operatorname{var} iate{s_{ijk}} + {e_{ijk}},with\,{e_{ijk}} \sim \hfill \\\,\,\,\,\,\,\,\,\,\,\,\,\,\,\,\,\,\,\,\,\,\,\,\,\,\,\,\,\,\,\,\,\,\,\,\,\,\,\,\,\,\,\,\,\,\,N(0,{\delta ^2}) \hfill \\ \end{gathered}$$

Between-cell model (Level 2)

2 $$\begin{gathered}{\beta _{0jk}} = {\gamma _0} + {u_{0j}} + {v_{0k}},with\,{u_0}j \sim \hfill \\\;\;\;\;\;\;\;\;\;\;\;\;N(0,{\delta ^2}),{v_{0k}} \sim N(0,{\delta ^2}) \hfill \\ \end{gathered}$$

Combined model:

3 $$\begin{gathered}{\text{Dependent}}\,{\text{variable}}{{\text{s}}_{ijk}} = {\gamma _0} + {\beta _1}Ag{e_{ijk}} + {\beta _2}Age_{ijk}^2 + \hfill \\\,\,\,\,\,\,\,\,\,\,\,\,\,\,\,\,\,\,\,\,\,\,\,\,\,\,\,\,\,\,\,\,\,\,\,\,\,\,\,\,\,\,\,\,\,\,\,\,\,{\beta _l}Covariate{s_{ijk}} + {u_{0j}} + {v_{0k}} + {e_{ijk}} \hfill \\ \end{gathered}$$

Where${\gamma }_{0}$indicates the intercepts; *β* indicates the coefficients of age, age-squared (assessing nonlinear relationship), and covariates; *i* denotes individuals within cohort *j* and period *k*; ${u}_{0j}$and ${v}_{0k}$ are the residual random effects of cohort *j* and period *k*, respectively; ${e}_{ijk }$is the random individual effect.${u}_{0j}$and ${v}_{0k}$ and ${e}_{ijk }$are assumed to be normally distributed with mean = 0 and a with-cell variance ${\delta }^{2}$ [36]. 

Dependent variables in the current study included old-age pension coverage (dichotomous), four dummy variables of eldercare responsibility (dichotomous), and filial piety score (continuous); Age was included in the HAPC model after centering around grand means to stabilize estimation and prevent bias resulting from the systematic variation in mean age across different cohorts [36]. Likewise, education level, as an ordinal categorical variable, was centered because higher education attainment substantially increased in younger cohorts. Marital status was included after creating two dummy variables: unmarried (yes/no) and married/partnership (yes/no). The coefficients of age, age-squared, and covariates were estimated using the restricted maximum likelihood approach and then transformed into odds ratios (ORs) to facilitate the interpretation of fixed effects, except for the filial piety score. The estimated probability of old-age pension coverage and primary eldercare responsibility and the predicted score of filial piety for age, period, and cohort effects are presented graphically. The HAPC model was analyzed using the ‘lme4’ package, and its model fitness was determined using the Bayesian information criterion (BIC). Marginal *R*^2^ was used to denote the variance of fixed effects only, conditional *R*^2^ to denote the variance of both fixed and random effects, and the intraclass correlation coefficient (ICC) for indicating the mean random effect variance [37]. The HAPC model with the variance of the random effect for period/cohort = 0 was further processed by including it as a fixed effect.

To ensure the reliability of the HAPC model, we also applied the generalized additive model (GAM) to disentangle the age/period/cohort effect. The GAM addresses the ‘identification problem’ by examining the nonlinear age, period, and cohort effects using a bivariate spline function that combines age and period to indirectly indicate the cohort effect [35]. Referring to Weigert et al. (2022) [35], we designed the following model:

4 $$\begin{gathered}{\text{log}}\left( {\frac{{{P_i}}}{{1 - {P_i}}}} \right) = {\beta _0} + {f_{ap}}\left( {ag{e_i},perio{d_i}} \right) + {\omega _i}, \hfill \\\,\,\,\,\,\,\,\,\,\,\,\,\,\,\,\,\,\,\,\,\,\,\,\,\,\,\,\,\,\,\,\,\,i = 1, \ldots ,n \hfill \\ \end{gathered}$$

Where ${P}_{i}$ denotes the probability of participating in the old-age pension scheme or the respective primary eldercare responsibility; ${\beta }_{0}$is the intercept; ${f}_{ap}\left({age}_{i}, { period}_{i}\right)$is the age-period two-dimensional nonlinear function; ${\omega }_{i}$is the linear/nonlinear effect of other covariates. Because the filial piety scores available only in two study periods cannot support the requirement of at least three knots for spline function, the corresponding GAM-based APC was unavailable.

The heatmap referring to the Lexis diagram with five years in the age group and one year in the cohort group was used to indicate the interrelation of age/period/cohort effect, and its specific effect was also graphically displayed [36]. The GAM-based APC model was analyzed using the ‘APCtools’ package, and its model fitness was represented by *R*^2^.

Subgroup analysis by registered residence (urban and rural) was further applied to all APC models. Missing values in the APC models were deleted. The sensitivity analysis was conducted by repeating all APC analyses after re-categorizing the cohort by 10-year interval or winsorizing the age group with a small sample size (participants aged 80–90 years only accounted for around 2%). Moreover, given that multidimensional filial piety currently emphasizes respect and reciprocity more than patrilineality and gender norms, we further excluded the patrilineality-related question (Offspring should respect father’s authority in any case) and gender norms-related question (Offspring should give birth to at least one male heir to preserve the family lineage) to reconstruct the filial piety score and repeated HAPC analysis.

## Results

### Descriptive characteristics

Among 66,182 participants (mean age: 48.8 years, females: 51.7%) with complete information on covariates in a total of six waves, 56,587 had complete data on old-age pension participation or primary eldercare responsibility concept in five waves from 2010 to 2017. The mean age, proportion of rural residents, higher education level, and old-age pension coverage of eligible participants gradually increased as the study period progressed (Table S1). The offspring-based and the government/self/offspring-shared eldercare concept dominated the primary eldercare responsibility (over 84%). Furthermore, the proportion of the offspring-based concept gradually declined over time. Table S2 displays the characteristics of 7,085 participants with complete information on filial piety score in 2006 and 2017. Females and rural residents accounted for 54.3% and 52.9%, respectively. The mean traditional filial piety score was 37.46 ± 4.66 in 2006 and 37.86 ± 5.34 in 2017.

### Hierarchical APC (HAPC) model for old-age pension coverage

**Fig. 1:**
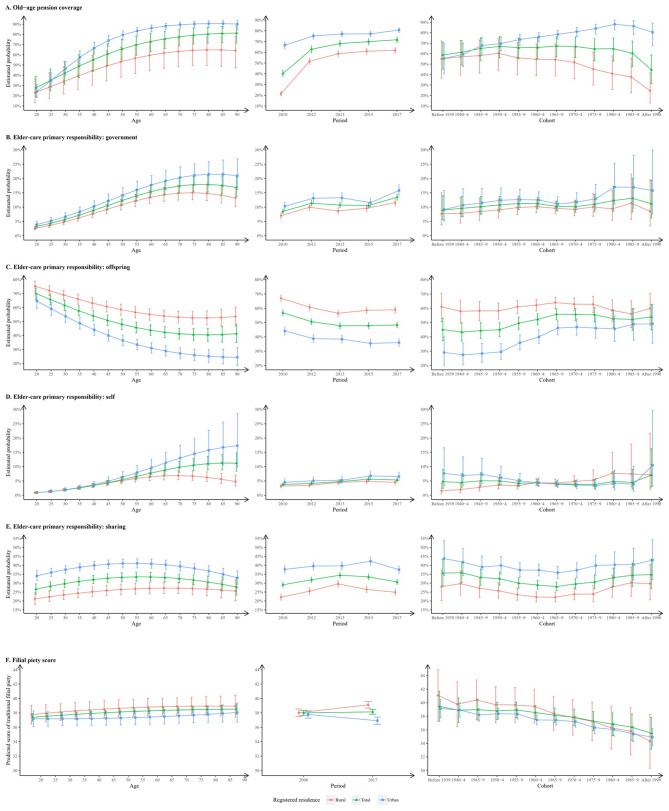
Age, period, and cohort effect of old-age pension coverage, primary eldercare responsibility concepts, and filial piety according to registered residence. **A**: Old-age pension coverage; **B**: Government-based eldercare; **C**: Offspring-based eldercare; **D**: Self-based eldercare; **E**: Sharing eldercare; **F**: Filial piety score. Green line with tringle points denotes total participants; Red line with circle points denotes rural residents; Blue line with square points denotes urban residents. The error bar represents the 95% confidence interval. The estimated probability or predicted score was calculated using hierarchical age-period-cohort models after adjusting for sex, ethnicity, (residence), education, and marital status

The results of the HAPC models (Table 1) show that old-age pension coverage increased with aging nonlinearly (odds ratio [OR]-age = 1.48, 95% CI: 1.39–1.57; OR-age^2^ = 0.95, 95% CI: 0.94–0.98) after adjusting for sex, ethnicity, residence, education, and marital status. The old-age pension coverage rate in rural residents was much lower than that in urban residents (OR-residence = 0.54; 95% CI: 0.52–0.57), and this urban-rural difference was exacerbated with aging (Fig. 1A). The estimated old-age pension coverage increased from 2010 to 2012, which is mainly attributed to the abrupt increase in coverage among rural residents. Thereafter, it maintained a parallel and slight growth to that in urban residents but remained an urban-to-rural gap of over 20 (%). Old-age pension coverage was maintained at approximately 60% among individuals born before the 1980s cohort, whereas it declined significantly among those born later. Moreover, it differed in registered residence. Old-age pension coverage continuously increased from the old to the young birth cohort, and the highest level was around the 1980–1984 cohort in urban residents, whereas that in rural residents decreased dramatically after the 1950–1954 cohort. The ICC indicates that the random effects of cohort and period explain 8% of the variance in old-age pension coverage.

**Table 1:** Results for the hierarchical age-period-cohort model of old-age pension coverage by residence

HAPC model†	Total (*n* = 55,541)	Urban (*n* = 22,460)	Rural (*n* = 33,081)
**Fixed effect, (OR (95% CI),** ***p*** **-value)**			
Intercept	2.20 (1.39–3.47), 0.001	1.73 (1.28–2.34), < 0.001	1.22 (0.66–2.25), 0.525
Age‡	1.48 (1.39–1.57), < 0.001	1.76 (1.64–1.89), < 0.001	1.36 (1.28–1.43), < 0.001
Age^2^	0.95 (0.94–0.98), < 0.001	0.92 (0.89–0.94), < 0.001	0.95 (0.93–0.98), 0.002
Sex (female vs. male)	0.99 (0.95–1.03), 0.649	1.00 (0.94–1.07), 0.995	1.01 (0.96–1.06), 0.785
Ethnicity (Han vs. non-Han)	1.28 (1.20–1.37), < 0.001	1.52 (1.34–1.73), < 0.001	1.22 (1.13–1.32), < 0.001
Registered residence (rural vs. region)	0.54 (0.52–0.57), < 0.001		
Education‡	1.54 (1.50–1.58), < 0.001	1.85 (1.78–1.92), < 0.001	1.34 (1.29–1.38), < 0.001
Marital status			
Unmarried vs. others	0.93 (0.84–1.04), 0.197	1.21 (1.01–1.43), 0.035	0.81 (0.70–0.93), 0.004
Married/partnership vs. others	1.19 (1.12–1.27), < 0.001	1.27 (1.14–1.42), < 0.001	1.12 (1.03–1.22), 0.01
**Random effects variance**			
Cohort	0.045	0.037	0.023
Period	0.238	0.061	0.449
ICC	0.08*	0.03*	0.13**
**Model fitness**			
Bayesian information criterion	63194.7	22122.8	40502.1
Marginal *R*^2^	0.146	0.175	0.051
Conditional *R*^2^	0.213	0.198	0.170

†Adjusted for sex, ethnicity, (residence), education, and marital status;

‡Age and education were centered around grand means.

*ICC is significant with *p*-value < 0.05; ** with *p*-value < 0.01.

OR: odds ratio; CI: confidence interval; ICC: intraclass correlation coefficient

### HAPC model for the primary eldercare responsibility

For the primary eldercare responsibility (Table 2), the effect of age was nonlinearly negative for the offspring-based concept (OR-age = 0.81, 95% CI: 0.79–0.84) but nonlinearly positive for the government-based (OR-age = 1.37, 95% CI: 1.33–1.41), self-based (OR-age = 1.55, 95% CI: 1.47–1.63), and sharing (OR-age = 1.03, 95% CI: 1.01–1.06) concepts. Rural residents were more likely to perceive the offspring-based concept (OR-residence = 1.99; 95% CI: 1.91–2.07), while less likely to deem the government-based (OR-residence = 0.53; 95% CI: 0.50–0.57), self-based (OR-residence = 0.52; 95% CI: 0.48–0.57), and sharing (OR-residence = 0.72; 95% CI: 0.69–0.75) concepts than in urban residents. Likewise, the urban-rural gap in the government-based, offspring-based, and self-based concepts grew with aging (Fig. 1B-D). Figure 1C shows the offspring-based concept gradually declined from 2011 to 2013 and then stabilized. For cohort effect, it increased gradually among those born between the 1940s and 1970s cohorts and stabilized thereafter. Comparatively, the other three forms of eldercare concepts almost flattened across the entire study period and birth cohort groups (Fig. 1B, C, E). However, there has been a marked increase in the government-based eldercare concept since the study year 2015 and the cohort of the 1980s, particularly among urban residents. The urban-rural difference in the trends of estimated probability in all four concepts by period and cohort group was almost similar. Note that the variance explained by the mean random effects of cohort and period was ≤ 1%. Their corresponding APC models with further inclusion of cohort as a fixed term showed that the results of the age effect remained similar but with a worse model fitness (Table S3).

**Table 2:** Results for the hierarchical age-period-cohort model of primary elder-care responsibility conception by residence

HAPC model†	Total (*n* = 56,332)		Urban (*n* = 22,808)		Rural (*n* = 33,524)	
**For the government**						
**Fixed effect, (OR (95% CI), ** ***p*** **-value)**						
Intercept	0.15 (0.12–0.18), < 0.001		0.13 (0.10–0.17), < 0.001		0.09 (0.07–0.11), < 0.001	
Age‡	1.37 (1.33–1.41), < 0.001		1.37 (1.31–1.44), < 0.001		1.37 (1.32–1.42), < 0.001	
Age^2^	0.95 (0.93–0.96), < 0.001		0.95 (0.93–0.97), < 0.001		0.94 (0.92–0.95), < 0.001	
Sex (female vs. male)	1.09 (1.03–1.15), 0.004		1.06 (0.98–1.14), 0.169		1.11 (1.02–1.21), 0.017	
Ethnicity (Han vs. non-Han)	1.07 (0.96–1.20), 0.198		1.26 (1.05–1.50), 0.012		0.97 (0.84–1.11), 0.658	
Registered residence (Rural vs. region)	0.53 (0.50–0.57), < 0.001					
Education‡	1.03 (1.00–1.07), 0.064		1.01 (0.97–1.06), 0.522		1.08 (1.02–1.14), 0.008	
Marital status						
Unmarried vs. others	1.54 (1.31–1.80), < 0.001		1.26 (1.01–1.58), 0.038		1.98 (1.58–2.49), < 0.001	
Married/partnership vs. others	0.95 (0.88–1.04), 0.296		0.94 (0.84–1.06), 0.342		0.96 (0.84–1.09), 0.505	
**Random effects variance**						
Cohort	0.002		0.006		0.000	
Period	0.027		0.026		0.030	
ICC	0.01*		0.01*		NA	
**Model fitness**						
Bayesian information criterion	35270.0		17683.7		17645.3	
Marginal *R*^2^	0.089		0.066		0.057	
Conditional *R*^2^	0.097		0.075		NA	
**For the offspring**						
**Fixed effect, (OR (95% CI)**, ***p*-value)**						
Intercept	0.81 (0.68–0.95), 0.011		0.89 (0.72–1.08), 0.237		1.54 (1.29–1.86), < 0.001	
Age‡	0.81 (0.79–0.84), < 0.001		0.75 (0.72–0.79), < 0.001		0.84 (0.82–0.87), < 0.001	
Age^2^	1.04 (1.02–1.05), < 0.001		1.04 (1.01–1.06), < 0.001		1.03 (1.01–1.05), < 0.001	
Sex (female vs. male)	1.11 (1.07–1.15), < 0.001		1.18 (1.11–1.25), < 0.001		1.07 (1.02–1.12), 0.003	
Ethnicity (Han vs. non-Han)	0.80 (0.75–0.85), < 0.001		0.70 (0.63–0.79), < 0.001		0.85 (0.78–0.91), < 0.001	
Registered residence (rural vs. region)	1.99 (1.91–2.07), < 0.001					
Education‡	0.79 (0.77–0.81), < 0.001		0.78 (0.76–0.81), < 0.001		0.79 (0.76–0.81), < 0.001	
Marital status						
Unmarried vs. others	0.80 (0.73–0.89), < 0.001		0.73 (0.63–0.85), < 0.001		0.79 (0.69–0.90), 0.001	
Married/partnership vs. others	0.94 (0.88–0.99), 0.031		0.94 (0.86–1.03), 0.184		0.95 (0.88–1.03), 0.227	
**Random effects variance**						
Cohort	0.010		0.016		0.007	
Period	0.019		0.017		0.023	
ICC	0.01*		0.01*		0.01*	
**Model fitness**						
Bayesian information criterion	73832.2		29424.2		44367.1	
Marginal *R*^2^	0.083		0.044		0.018	
Conditional *R*^2^	0.091		0.053		0.027	
**For the self**						
**Fixed effect, (OR (95% CI)**, ***p*-value)**						
Intercept	0.06 (0.04–0.07), < 0.001		0.06 (0.04–0.08), < 0.001		0.03 (0.02–0.04), < 0.001	
Age‡	1.55 (1.47–1.63), < 0.001		1.51 (1.51–1.83), < 0.001		1.41 (1.33–1.49), < 0.001	
Age^2^	0.94 (0.92–0.96), < 0.001		0.95 (0.91–0.98), 0.003		0.91 (0.89–0.94), < 0.001	
Sex (female vs. male)	1.00 (0.92–1.07), 0.898		0.90 (0.81–1.00), 0.053		1.11 (0.99–1.24), 0.065	
Ethnicity (Han vs. non-Han)	1.03 (0.89–1.19), 0.697		1.00 (0.80–1.26), 0.972		1.05 (0.87–1.27), 0.623	
Registered residence (rural vs. region)	0.52 (0.48–0.57), < 0.001					
Education‡	1.00 (0.96–1.05), 0.956		1.03 (0.98–1.08), 0.298		0.99 (0.92–1.08), 0.899	
Marital status						
Unmarried vs. others	1.39 (1.07–1.79), 0.013		1.11 (0.74–1.66), 0.622		1.87 (1.31–2.67), 0.001	
Married/partnership vs. others	1.37 (1.22–1.54), < 0.001		1.28 (1.10–1.50), 0.001		1.43 (1.18–1.73), < 0.001	
**Random effects variance**						
Cohort	0.006		0.036		0.004	
Period	0.023		0.024		0.025	
ICC	0.01*		< 0.01*		0.01*	
**Model fitness**						
Bayesian information criterion	22463.1		11232.7		11216.1	
Marginal *R*^2^	0.145		0.168		0.081	
Conditional *R*^2^	0.153		0.183		0.089	
**For the sharing**						
**Fixed effect, (OR (95% CI)**, ***p*-value)**						
Intercept	0.56 (0.49–0.64), < 0.001		0.53 (0.46–0.62), < 0.001		0.40 (0.33–0.48), < 0.001	
Age‡	1.03 (1.01–1.06), 0.008		1.02 (1.00–1.04), 0.088		1.05 (1.02–1.09), 0.003	
Age^2^	0.98 (0.96–0.99), 0.002		0.97 (0.96–0.98), < 0.001		0.98 (0.97–1.00), 0.124	
Sex (female vs. male)	0.86 (0.83–0.89), < 0.001		0.86 (0.81–0.90), < 0.001		0.87 (0.83–0.91), < 0.001	
Ethnicity (Han vs. non-Han)	1.26 (1.17–1.34), < 0.001		1.31 (1.16–1.47), < 0.001		1.23 (1.13–1.34), < 0.001	
Registered residence (rural vs. region)	0.72 (0.69–0.75), < 0.001					
Education‡	1.27 (1.24–1.30), < 0.001		1.24 (1.21–1.28), < 0.001		1.30 (1.25–1.34), < 0.001	
Marital status						
Unmarried vs. others	1.05 (0.95–1.16), 0.373		1.17(1.02–1.35), 0.024		0.94 (0.82–1.09), 0.434	
Married/partnership vs. others	0.98 (0.92–1.04), 0.548		1.00 (0.91–1.09), 0.969		0.99 (0.91–1.08), 0.803	
**Random effects variance**						
Cohort	0.004		0.000		0.007	
Period	0.008		0.004		0.016	
ICC	< 0.01		NA		0.01*	
**Model fitness**						
Bayesian information criterion	69816.6		30555.2		39292.7	
Marginal *R*^2^	0.040		0.020		0.014	
Conditional *R*^2^	0.043		NA		0.020	

†Adjusted for sex, ethnicity, (residence), education, and marital status;

‡Age and education were centered around grand means.

*ICC is significant with *p*-value < 0.05; ** with *p*-value < 0.01.

OR: odds ratio; CI: confidence interval; ICC: intraclass correlation coefficient

**Table 3:** Results for the hierarchical age-period-cohort model of traditional filial piety score by residence

**HAPC model†**		**Total (** *n* ** = 7,085)**		**Urban (** *n* ** = 3,339)**		**Rural (** *n* ** = 3,746)**
**Fixed effect, (Estimate (SE)**, ***p*-value)**						
Intercept		37.87 (0.32), < 0.001		37.69 (0.65), < 0.001		38.51 (0.69), < 0.001
Age‡		0.18 (0.05), < 0.001		0.10 (0.08), 0.174		0.20 (0.08), 0.015
Age^2^		-0.02 (0.03), 0.418		0.02 (0.04), 0.535		-0.03 (0.04), 0.424
Sex (female vs. male)		0.33 (0.12), 0.005		0.46 (0.17), 0.007		0.22 (0.17), 0.19
Ethnicity (Han vs. non-Han)		-1.01 (0.24), < 0.001		-1.30 (0.41), 0.001		-0.80 (0.30), 0.008
Registered residence (rural vs. region)		0.77 (0.13), < 0.001				
Education‡		-0.63 (0.07), < 0.001		-0.49 (0.10), < 0.001		-0.78 (0.11), < 0.001
Marital status						
Unmarried vs. others		0.12 (0.32), 0.701		0.01 (0.44), 0.984		0.12 (0.47), 0.799
Married/partnership vs. others		0.26 (0.22), 0.220		0.53 (0.30), 0.078		-0.01 (0.30), 0.975
**Random effects variance**						
Cohort		0.000		0.000		0.040
Period		0.002		0.351		0.580
ICC		NA		NA		0.02*
**Model fitness**						
Bayesian information criterion		42855.3		20100.4		22766.7
Marginal *R*^2^		0.047		0.022		0.031
Conditional *R*^2^		NA		NA		0.055

†Adjusted for sex, ethnicity, (residence), education, and marital status;

‡Age and education were centered around grand means.

*ICC is significant with *p*-value < 0.05.

SE: standard error; ICC: intraclass correlation coefficient

### HAPC model for the traditional filial piety score

The age effect on filial piety score (Table 3) was significant, but its magnitude was small, with an increase of 0.18 (Standard error [SE] = 0.05) per year of age. The traditional filial piety score in rural residents was much higher than that in urban residents (coefficient = 0.77, SE = 0.13); however, the urban-rural difference in the predicted filial piety score by age group was not significant (Fig. 1F). In rural residents, the predicted filial piety score decreased from 2006 to 2017 and decreases as the cohort was younger, with the random effects of period and cohort explaining a 2% variance. Given that the random effect of the cohort was equivalent to zero in total and urban residents, we replaced it as a fixed term and found the age effect to be non-significant (coefficient = -0.48, SE = 0.35) with the random effect of the period explaining 2% variance in total residents (Table S3); however, the zero random effect of time in rural residents indicated that mixed effects model was inappropriate. We further matched a linear regression model and found that age, period, and cohort effects were non-significant (Table S4).

Before conducting the adjusted HAPC model, the crude HAPC model without adjusting for any covariates was also conducted (Table S5). The results were similar, but the adjusted model improved fitness.

### Generalized additive model (GAM) based APC analysis

The interaction between age and period effects is displayed using a heatmap (Figure S1). The GAM-based APC model calculated the estimated marginal ORs of age, period, and cohort effect (Figure S1 and Table S6). For old-age pension coverage, older age groups and recent periods showed the highest probability of participating in the old-age pension scheme (Figure S1A). The tendency for old-age pension coverage increased with age, peaking at the age of 72 years (OR = 1.55) and then continuously decreasing. The marginal ORs of the cohort effect display a reversed U-shape, with individuals born in 1942 having the highest probability of participating in the old-age pension scheme (OR = 1.53). However, the marginal OR continuously decreased as the birth cohort grew younger since the birth cohort of 1942.

For the primary eldercare responsibility concept, older age groups across period groups were more likely to perceive the government-based concept (Figure S1B) with the highest likelihood around age 76 (OR = 1.66) and self-based (Figure S1D) concepts with the highest chance around age 75 (OR = 2.10), whereas they were less likely to perceive the offspring-based concept (Figure S1C) with the lowest probability around age 71 (OR = 0.61). The older adults aged between 60 and 80 in the middle study period had a higher likelihood of the sharing concept (Figure S1E), with the highest chance around age 69 (OR = 1.17). Likewise, cohort effects showed that individuals born between the 1930s and 1950s were less likely to perceive offspring-based eldercare but more likely to perceive the other three eldercare forms. The max/minimum OR ratio showed that age and cohort effects dominated the APC effects. Additionally, the urban-rural difference indicates that the period effect on old-age pension coverage and the primary eldercare responsibility concept was mainly attributed to that in rural residents (Figure S2).

### Sensitivity analysis

The results of sensitivity analysis after re-categorizing as a 10-year interval in the cohort or winsorizing the 80–90 years as 80 years in age were identical to the main results with a slightly improved model fitness (data not shown). The effect of age and period effect on filial piety score after removing the patrilineality and gender norms-related questions was non-significant. Similar to the main results, the youngest cohort (the 1990s) reported a lower score (Table S7 and Figure S3).

## Discussion

This is the first study to comprehensively examine age, period, and cohort trends in old-age pension coverage, primary eldercare responsibility, and traditional filial piety in China from 2006 to 2017. We found that old-age pension coverage increased substantially across age and period groups; however, it remained low in young and middle-aged adults in rural areas. The offspring-based eldercare concept is continuously weakened over aging and time and is partially transferred into government-based and self-reliant eldercare in older adults. However, the offspring-based eldercare concept still prevails among rural residents. Consistent with the initial hypotheses, the concept of government-based and sharing of primary eldercare responsibility popularized in young adults born after the 1980s. Moreover, traditional filial piety is gradually diluted in this population.

Our study, consistent with a previous related study also using CGSS data [15], observed a remarkable increase in old-age pension coverage over time. This increase was driven by the Chinese pension reform, which launched the NRPS for the rural population in 2009 and the URPS for non-employed urban residents in 2011 [13]. The NRPS successfully involved rural residents in the social pension schemes promoted by the government quota subsidy [13], which consequently explained why old-age pension coverage increased abruptly in rural residents from 2010 to 2012. However, the URPS is a supplementary pension scheme that covers only non-employed urban adults [13]. Therefore, old-age pension coverage for urban residents started at a higher level but increased slowly from 2010 to 2017. Furthermore, our study revealed the urban-rural difference in old-age pension coverage intensified as the birth cohort was younger. Young and middle-aged urban residents are commonly employed in urban enterprises, with 20%/12% of their monthly salary paid by employers/self-employees and 8% by employees themselves mandatorily to construct the specialized old-age pension systems for urban employees [13]. However, old-age pension coverage in rural young and middle-aged adults was adversely affected by a range of factors, including their remote eldercare needs and persistent offspring-based eldercare concept, higher population mobility to urban areas, and lower durable assets [38]. Thus, improving the coverage of old-age pension in this population to ensure equity is required [39, 40]. 

Similarly, our study and those of Zhao et al. (2021) [15] and Wang et al. (2009) [20] consistently indicated that the traditional offspring-based eldercare concept is prevailing but partially replaced with government-based and self-reliance concepts, particularly in old-age urban residents. The reason for this social concept change involves multiple dimensions. At the macro level, personal disposable property accumulates as economic development progresses, and the involvement of old-age pension schemes has made traditional family finance dependence more egalitarian [41]. The replacement rate of government/institution and enterprise employee pensions was approximately 60% in 2015, which is even higher than the average earnings of young adults in some cities [40]. Consequently, older adults gained more autonomy in choosing the eldercare pattern. Additionally, social injustice may influence parents’ concerns about relying on children to sustain their old life and expect government-based eldercare to reconcile this concern instead [22]. At the micro level, within the excessive migrations from villages or small cities to megacities and related high living expenses, older parents live alone and gradually decrease their expectation of eldercare support from children [18, 20]; the practice and willingness of adult children to fulfill eldercare for parents become increasingly difficult [18, 42]. Therefore, more government and community resources have been involved in supporting the increasing eldercare needs as the offspring-based eldercare concept is diluted.

Differing from the multifarious concepts of primary eldercare responsibility in older adults, the youngest adults (aged 20–25 years) tend to perceive that offspring should undertake primary eldercare responsibility instead of the government and the elders themselves. Moreover, the findings of our study indicated that the traditional piety score in the younger birth cohort gradually declined. Adult children born in the 1970s and 1980s were more influenced by economic independence and individualism as the Reform and Opening-up Policy was issued; those born in the 1990s were almost the only child in the family because of the One-Child Policy and were more self-centered without less concern about the eldercare needs of parents [43]. Conversely, a few investigators found that single-child is more likely to support their parents and reside in the same city in the future [44], particularly those with higher education attainment [8, 24]. A few studies have explored the evolution process of traditional filial piety and found that what matters about this conventional Chinese norm is note the quantified level but the expanded connotation [24, 44, 45]. Traditional filial piety emphasizes patrilineal values (authoritarian) and obedience [44, 45]; however, it has evolved to address reciprocity, respect, and caring themes [45]. Regardless, the role of filial piety in the future pension policy is ‘ever-present’ because it carries a unique emotional bond between children and parents.

Our study also explored potential sociodemographic factors affecting old-age pension coverage, the concept of primary eldercare responsibility, and traditional filial piety. Ethnic minority is a risk factor for old-age pension coverage and sharing eldercare responsibility but a protective factor for the offspring-based concept and traditional filial piety, mainly attributed to specific cultural values, residence patterns, and education level [46]. We also found that females are more likely to support offspring-based eldercare and perceive more traditional filial piety than males; however, this does not mean that females are more obedient to conventional Chinese values. In contrast, they are more likely to challenge traditional Chinese values and benefit more by removing this persecution [24]. This can be affirmed by the fact that conventional son preference has declined remarkably since the One-Child Policy and the increased social involvement of females in current Chinese society [44, 47]. Indeed, females undertake more responsibility in internal family affairs and can provide better care when the eldercare needs of old parents occur, particularly for emotional support [47]. 

### Policy implications

With a rapidly aging population and substantially declining fertility, China is encountering enormous pressures on eldercare needs [18, 29]. Children’s filial piety beliefs and behaviors play essential roles in the psychological well-being of older parents [15, 30]. Healthy aging, focusing on developing and maintaining the physical and mental capacities of older people, is now the core content of the national strategic development framework [13, 18, 29]; our study provides the following policy implications:

Diversifying primary eldercare responsibility concepts require more involvement from the government and social sources. The national number of eldercare facilities increased from 40,000 in 2010 to 220,000 in 2020, and the number of eldercare beds also tripled to 7.9 million [48]. In parallel, serial of problems and challenges are emerging, including varying degrees of regulation and quality of eldercare, inconvenient location, and shortage of nurse workers [18, 29]. Although the private sector provides relatively qualified eldercare, its average estimated cost accounts for approximately 70% of the average pension per month [29]. Thus, well-supervised, convenient, and affordable public eldercare systems are required. Moreover, allowing private institutions to achieve low profit via appropriate scale-up and ensuring the differentiation of eldercare services to satisfy various eldercare needs should be encouraged [49]. However, the offspring-based care responsibility concept in our study still dominates, which indicates that the family-based eldercare pattern will remain the mainstream form for a long time. Correspondingly, multidimensional supplemental measures, including community-based eldercare, integration of treatment and convalescence, and long-term care insurance, should be strengthened and centered on family-based eldercare [50]. 

The significant urban-rural difference requires more precise aging policies for older rural residents. Currently, the government allocates more eldercare resources to senior urban residents [18]. Moreover, the expectation and willingness of older rural residents to live in nursing homes remained sluggish [42]. Zhang et al. (2021) recruited 515 urban and 429 rural older residents and found that the psychological needs of urban residents were mainly influenced by neighborhood support, whereas children’s support was the primary determinant for rural residents [51]. In addition, urban single-child parents mostly preferred pension insurance to support their old life, whereas rural counterparts favored personal savings and family support [27]. The demand for long-term eldercare in older rural residents was also superior to that in urban residents [52]. Conventional strategies for addressing urban residents’ eldercare needs do not apply to rural residents. Recently, the Chinese government has encouraged migrants to return to their hometowns for business initiation and employment during rural vitalization [53], which may be a sustainable solution for the long-term eldercare of old rural residents.

The gradually declining filial piety in the younger cohorts requires more cultivation in society and schools. Financial and emotional support from adult children benefits the well-being of older parents [15, 30, 45]. Promoting filial piety effectively relieves the eldercare burden on the government and improves social and family harmony [11, 12, 14, 15]. However, the promoting process should be sensible for the essence and dross of traditional filial piety and develop contemporary filial piety that address respect, devotion, equality, and reciprocity instead of obedience and authorism [45]. Education policy and practice have been well documented to resist the erosion of Chinese modernization on filial piety [12]. Moreover, filial piety through voluntary contracts has emerged to better assist older adults with their eldercare needs [54]. Therefore, enhancing contemporary filial piety education as an adjunct to appropriate social legislation is warranted.

### Strength and limitations

The major strength of this study is the large sample size based on nationally representative data from the CGSS. Utilizing data from over 60,000 participants from 2006 to 2017, this study provides an overview of the transition of old-age pension coverage, the primary eldercare responsibility concept, and traditional filial piety and its urban-rural difference in China. Moreover, time trends and intergenerational impact were mutually examined using two advanced statistical methods (mixed effect model and GAM) to enhance robustness. The results regarding the age, period, and cohort effects on old-age pension coverage and the primary eldercare responsibility concept were similar except that the GAM method provided more details on the nonlinear effect of age, period, and cohort.

Several limitations should be noted. The measurement of primary eldercare responsibility and traditional filial piety was based on simple questions rather than validated and systematic questionnaires; however, standardized tools are yet to be widely used [25, 55]. A well-developed and validated questionnaire to assess eldercare responsibility and filial concept is required, which is imperative to understand the common social eldercare concept in the current aging society. Defining urban and rural residents on the basis of registered residence fails to consider internal migrants. Some people are registered as rural residents but live in urban areas for long periods and are affected more by urban culture [56]. Finally, the selection bias of the repeated cross-sectional design may obscure the actual age, period, and cohort effects.

## Conclusion

Old-age pension coverage has increased remarkably during the last decade in China; however, its enhancement in rural young and middle-aged adults is warranted. Traditional filial piety is gradually diluted in adults born in the younger cohort, and the offspring-based eldercare concept is continuously weakened in older adults, particularly in urban areas. Self-reliance and government-led eldercare, filial piety education strength, and related urban-rural variations require more attention in future healthy aging policies.

### Electronic supplementary material

Below is the link to the electronic supplementary material.

Supplementary Material 1

Supplementary Material 2

## Data Availability

The datasets used and/or analyzed in the current study are available from the official website of CGSS, http://www.cnsda.org/index.php?r=projects/view&id=94525591.

## Abbreviations

APC: age-period-cohort model
HAPC: hierarchical age-period-cohort model
CGSS: Chinese General Social Survey
GAM: generalized additive model
OR: odds ratio
CI: confidence interval
SE: Standard error
