# Supplementary material for: The transition of eldercare responsibility and traditional filial piety concepts and its urban-rural differences in China: an age-period-cohort analysis from 2006 to 2017
Source: BMC Public Health. 2024 Jun 22;24:1669. doi: 10.1186/s12889-024-19175-5 (PMC11193898; doi:10.1186/s12889-024-19175-5)

**Supplementary Figure S1**. Heatmaps of the estimated tensor product surface and its 95% confidence interval for the old-age pension coverage and primary eldercare responsibility concepts

**A**: Old-age pension coverage; **B**: Government-based eldercare concept; **C**: Offspring-based eldercare concept; **D**: Self-based eldercare concept; **E**: sharing eldercare concept.

Effects are averaged over a one-year period interacted with five-year age; Exponentiated effects lower than 0.1 are trimmed to 0.1. The estimated effect was calculated by generalized additive models after adjusting for sex, ethnicity, residence, education, and marital status.


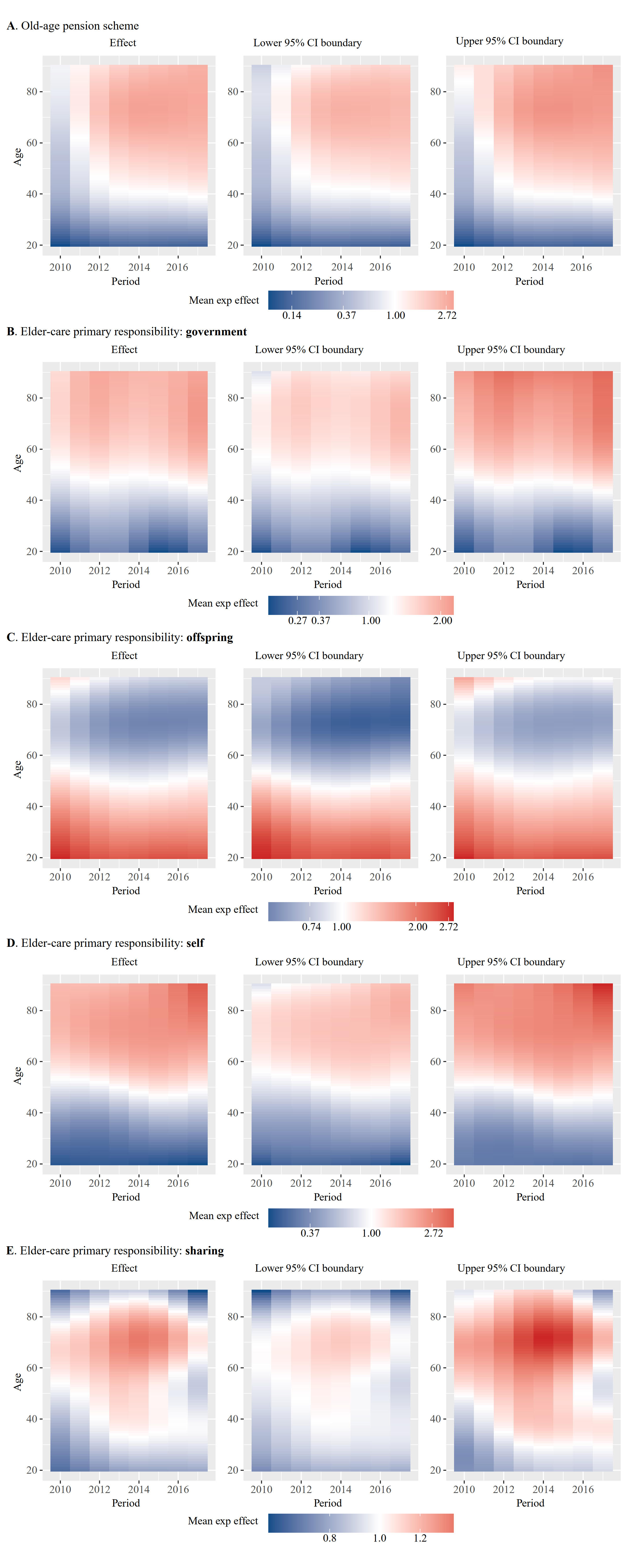


**Supplementary Figure S2**. Estimated marginal odds ratios of age, period, and cohort for the old-age pension coverage and primary eldercare responsibility concepts by residence

**A**: Total; **B**: Urban; **C**: Rural.

Colored lines denote different outcomes. The estimated effect was calculated by generalized additive models after adjusting for sex, ethnicity, (residence), education, and marital status.


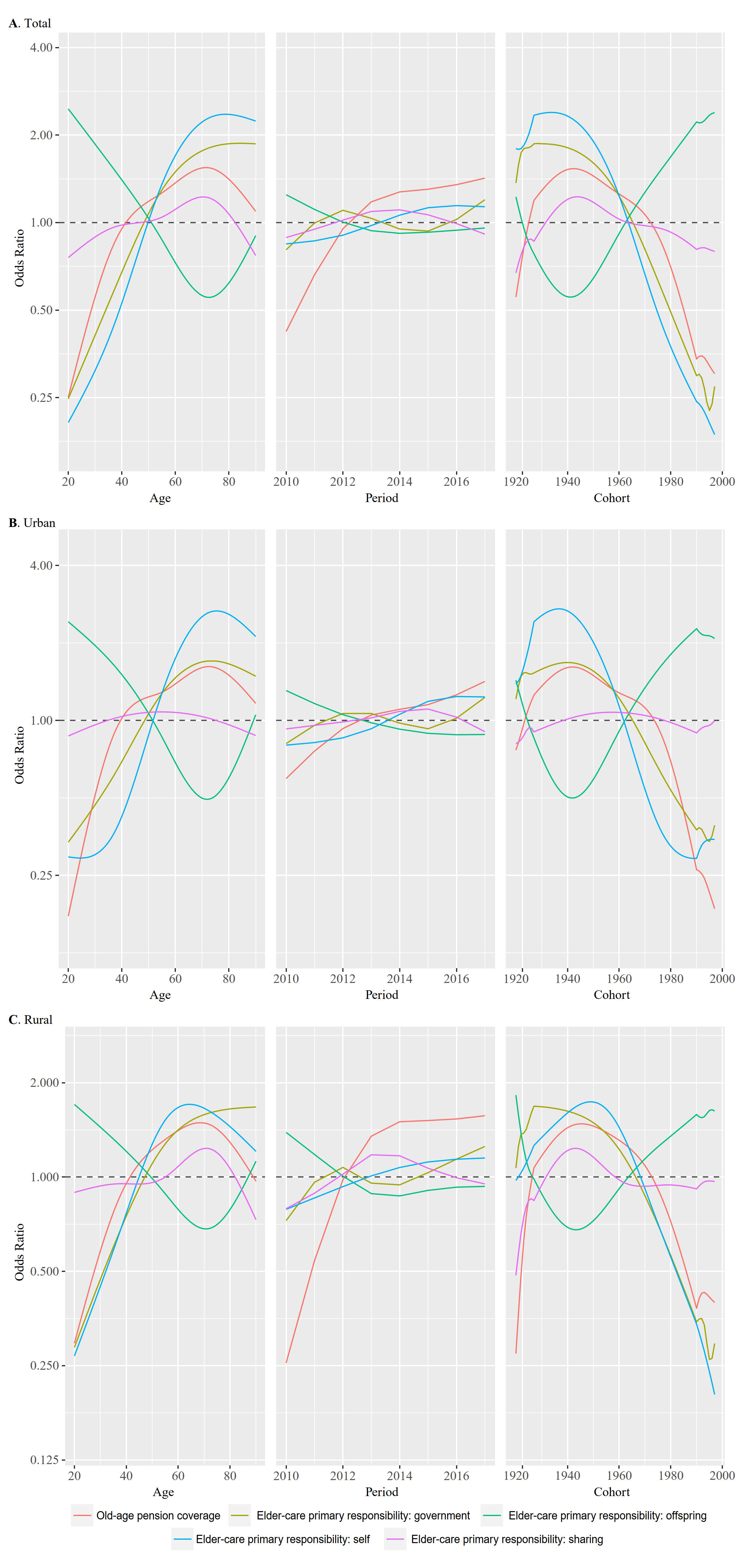


**Supplementary Figure S3**. Age, period, and cohort effect of the filial piety after removing questions related to patrilineality and gender norms by registered residence

Green line with tringle points denotes total participants; Red line with circle points denotes rural residents; Blue line with square points denotes urban residents. The error bar presents the 95% confidence interval. The estimated probability or predicted score was calculated by hierarchical age-period-cohort models after adjusting for sex, ethnicity, (residence), education, and marital status.


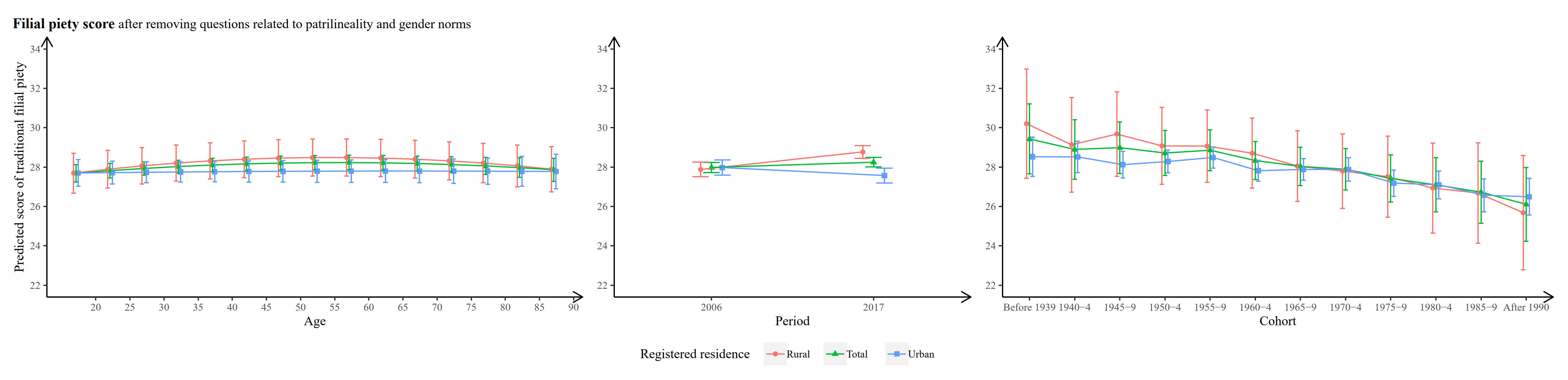

Supplement: Supplementary file 1 — Supplementary Material 1 [file 12889_2024_19175_MOESM1_ESM.doc]
