# Supplementary material for: The transition of eldercare responsibility and traditional filial piety concepts and its urban-rural differences in China: an age-period-cohort analysis from 2006 to 2017
Source: BMC Public Health. 2024 Jun 22;24:1669. doi: 10.1186/s12889-024-19175-5 (PMC11193898; doi:10.1186/s12889-024-19175-5)
Supplement: Supplementary file 2 — Supplementary Material 2 [file 12889_2024_19175_MOESM2_ESM.doc]

| **Table S1**. Characteristics of participants with complete information on old-age pension participation or eldercare responsibility concept | | | | | | |
| --- | --- | --- | --- | --- | --- | --- |
|  | **All (n = 56,587)** | **2010 (n = 10,953)** | **2012 (n = 11,525)** | **2013 (n = 11,127)** | **2015 (n = 10,695)** | **2017 (n = 12,287)** |
| **Age, yeas (mean ± SD)** | 49.74 ± 16.03 | 47.77 ± 15.29 | 49.33 ± 15.82 | 49.19 ± 15.91 | 50.86 ± 16.41 | 51.39 ± 16.40 |
| **Sex (%)** |  |  |  |  |  |  |
| Female | 29025 (51.3%) | 5687 (51.9%) | 5615 (48.7%) | 5535 (49.7%) | 5680 (53.1%) | 6508 (53%) |
| Male | 27562 (48.7%) | 5266 (48.1%) | 5910 (51.3%) | 5592 (50.3%) | 5015 (46.9%) | 5779 (47%) |
| **Ethnicity (%)** |  |  |  |  |  |  |
| Non-Han | 4743 (8.4%) | 1058 (9.7%) | 985 (8.5%) | 947 (8.5%) | 834 (7.8%) | 919 (7.5%) |
| Han nationality | 51844 (91.6%) | 9895 (90.3%) | 10540 (91.5%) | 10180 (91.5%) | 9861 (92.2%) | 11368 (92.5%) |
| **Registered residence (%)** |  |  |  |  |  |  |
| Urban | 22907 (40.5%) | 5038 (46%) | 4942 (42.9%) | 4445 (39.9%) | 3913 (36.6%) | 4569 (37.2%) |
| Rural | 33680 (59.5%) | 5915 (54%) | 6583 (57.1%) | 6682 (60.1%) | 6782 (63.4%) | 7718 (62.8%) |
| **Education (%)** |  |  |  |  |  |  |
| Primary school and lower | 20848 (36.8%) | 4116 (37.6%) | 4307 (37.4%) | 4039 (36.3%) | 4079 (38.1%) | 4307 (35.1%) |
| Middle school | 16327 (28.9%) | 3273 (29.9%) | 3281 (28.5%) | 3265 (29.3%) | 3039 (28.4%) | 3469 (28.2%) |
| High school | 10070 (17.8%) | 1977 (18%) | 2103 (18.2%) | 2019 (18.1%) | 1857 (17.4%) | 2114 (17.2%) |
| University and higher | 9342 (16.5%) | 1587 (14.5%) | 1834 (15.9%) | 1804 (16.2%) | 1720 (16.1%) | 2397 (19.5%) |
| **Marital status (%)** |  |  |  |  |  |  |
| Unmarried | 4708 (8.3%) | 862 (7.9%) | 840 (7.3%) | 929 (8.3%) | 948 (8.9%) | 1129 (9.2%) |
| Married/partnership | 45591 (80.6%) | 8992 (82.1%) | 9395 (81.5%) | 9022 (81.1%) | 8536 (79.8%) | 9646 (78.5%) |
| Divorced/widowed | 6288 (11.1%) | 1099 (10%) | 1290 (11.2%) | 1176 (10.6%) | 1211 (11.3%) | 1512 (12.3%) |
| **Old-age pension participation (%)** |  |  |  |  |  |  |
| No | 19581 (34.6%) | 5901 (53.9%) | 3978 (34.5%) | 3374 (30.3%) | 3091 (28.9%) | 3237 (26.3%) |
| Yes | 35960 (63.5%) | 4696 (42.9%) | 7368 (63.9%) | 7562 (68%) | 7464 (69.8%) | 8870 (72.2%) |
| **Elder-care primary responsibility (%)** |  |  |  |  |  |  |
| Government | 5661 (10%) | 836 (7.6%) | 1196 (10.4%) | 1074 (9.7%) | 1022 (9.6%) | 1533 (12.5%) |
| Offspring | 29038 (51.3%) | 6319 (57.7%) | 5968 (51.8%) | 5507 (49.5%) | 5256 (49.1%) | 5988 (48.7%) |
| Self | 3050 (5.4%) | 457 (4.2%) | 545 (4.7%) | 590 (5.3%) | 688 (6.4%) | 770 (6.3%) |
| Sharing | 18583 (32.8%) | 3297 (30.1%) | 3793 (32.9%) | 3933 (35.3%) | 3635 (34%) | 3925 (31.9%) |
| SD: standard deviation. | | | | | | |

| **Table S2**. Characteristics of participants with complete information on traditional filial piety score | | | |
| --- | --- | --- | --- |
|  | **All (n = 7,085)** | **2006 (n = 3,112)** | **2017 (n = 3,973)** |
| **Age, yeas (mean ± SD)** | 47.64 ± 15.55 | 43.07 ± 12.96 | 51.21 ± 16.45 |
| **Sex (%)** |  |  |  |
| Female | 3844 (54.3%) | 1710 (54.9%) | 2134 (53.7%) |
| Male | 3241 (45.7%) | 1402 (45.1%) | 1839 (46.3%) |
| **Ethnicity (%)** |  |  |  |
| Non-Han | 448 (6.3%) | 162 (5.2%) | 286 (7.2%) |
| Han nationality | 6637 (93.7%) | 2950 (94.8%) | 3687 (92.8%) |
| **Registered residence (%)** |  |  |  |
| Urban | 3339 (47.1%) | 1840 (59.1%) | 1499 (37.7%) |
| Rural | 3746 (52.9%) | 1272 (40.9%) | 2474 (62.3%) |
| **Education (%)** |  |  |  |
| Primary school and lower | 2259 (31.9%) | 912 (29.3%) | 1347 (33.9%) |
| Middle school | 2163 (30.5%) | 1033 (33.2%) | 1130 (28.4%) |
| High school | 1457 (20.6%) | 763 (24.5%) | 694 (17.5%) |
| University and higher | 1206 (17%) | 404 (13%) | 802 (20.2%) |
| **Marital status (%)** |  |  |  |
| Unmarried | 709 (10%) | 337 (10.8%) | 372 (9.4%) |
| Married/partnership | 5701 (80.5%) | 2596 (83.4%) | 3105 (78.2%) |
| Divorced/widowed | 675 (9.5%) | 179 (5.8%) | 496 (12.5%) |
| **Filial piety score (mean ± SD)†** | 37.69 ± 5.05 | 37.46 ± 4.66 | 37.86 ± 5.34 |
| ^†^Filial piety score was assessed by seven questions with seven Likert-scale options; its total score ranges from 7 to 49, with a higher score indicating more traditional filial piety. SD: standard deviation. | | | |

| **Table S3**. Results for the hierarchical age-period-cohort model without the random-effect of cohort | | | | |
| --- | --- | --- | --- | --- |
| **HAPC model†** | **The government-based eldercare responsibility in rural residents (n = 33,524)** | **The sharing eldercare responsibility in rural residents (n = 22,808)** | **The filial piety score in total (n = 7,085)** | **The filial piety score in urban residents (n = 3,339)** |
| **Fixed effect** | **OR (95% CI), *p*-value** | **OR (95% CI), *p*-value** | **Estimate (SE), *p*-value** | **Estimate (SE), *p*-value** |
| Intercept | 0.07 (0.03 – 0.14), <0.001 | 0.69 (0.44 – 1.08), 0.108 | 39.02 (1.19), <0.001 | 39.29 (0.85), <0.001 |
| Age‡ | 1.43 (1.10 – 1.86), 0.008 | 1.01 (0.88 – 1.17), 0.839 | -0.48 (0.35), 0.190 | -0.62 (0.16), <0.001 |
| Age^2^ | 0.95 (0.91 – 1.00), 0.042 | 0.95 (0.92 – 0.97), <0.001 | 0.06 (0.04), 0.129 | 0.07 (0.05), 0.171 |
| Cohort |  |  |  |  |
| 1940-4 vs. before 1939 | 1.02 (0.76 – 1.35), 0.911 | 0.92 (0.75 – 1.12), 0.417 | -0.58 (0.45), 0.203 | -0.21 (0.56), 0.707 |
| 1945-9 vs. before 1939 | 1.11 (0.77 – 1.60), 0.578 | 0.82 (0.64 – 1.05), 0.121 | -0.48 (0.55), 0.379 | -0.94 (0.57), 0.100 |
| 1950-4 vs. before 1939 | 1.19 (0.75 – 1.88), 0.457 | 0.85 (0.63 – 1.15), 0.304 | -0.68 (0.68), 0.320 | -0.75 (0.61), 0.216 |
| 1955-9 vs. before 1939 | 1.31 (0.75 – 2.28), 0.338 | 0.77 (0.54 – 1.10), 0.146 | -0.53 (0.82), 0.524 | -0.80 (0.66), 0.228 |
| 1960-4 vs. before 1939 | 1.36 (0.70 – 2.65), 0.370 | 0.76 (0.50 – 1.16), 0.211 | -0.92 (0.99), 0.357 | -1.68 (0.71), 0.019 |
| 1965-9 vs. before 1939 | 1.27 (0.59 – 2.76), 0.537 | 0.72 (0.45 – 1.16), 0.174 | -1.33 (1.14), 0.251 | -1.70 (0.76), 0.025 |
| 1970-4 vs. before 1939 | 1.22 (0.50 – 2.96), 0.665 | 0.76 (0.45 – 1.30), 0.32 | -1.62 (1.29), 0.221 | -1.92 (0.80), 0.016 |
| 1975-9 vs. before 1939 | 1.34 (0.48 – 3.73), 0.572 | 0.85 (0.47 – 1.55), 0.601 | -2.17 (1.47), 0.149 | -2.80 (0.85), 0.001 |
| 1980-4 vs. before 1939 | 1.24 (0.39 – 4.01), 0.715 | 0.87 (0.45 – 1.69), 0.675 | -2.63 (1.63), 0.118 | -3.10 (0.89), <0.001 |
| 1985-9 vs. before 1939 | 1.54 (0.41 – 5.83), 0.523 | 0.87 (0.42 – 1.82), 0.713 | -3.07 (1.80), 0.098 | -3.75 (0.95), <0.001 |
| After 1990 vs. before 1939 | 1.12 (0.25 – 5.06), 0.886 | 0.97 (0.43 – 2.20), 0.937 | -3.97 (2.03), 0.061 | -4.20 (1.01), <0.001 |
| Sex (female vs. male) | 1.11 (1.02 – 1.21), 0.015 | 0.85 (0.81 – 0.90), <0.001 | 0.340 (0.12), 0.005 | 0.47 (0.17), 0.006 |
| Ethnicity (Han vs. non-Han) | 0.97 (0.84 – 1.11), 0.652 | 1.31 (1.17 – 1.47), <0.001 | -0.98 (0.24), <0.001 | -1.31 (0.41), 0.001 |
| Registered residence (rural vs. region) |  |  | 0.75 (0.14), <0.001 |  |
| Education‡ | 1.07 (1.02 – 1.14), 0.013 | 1.24 (1.21 – 1.28), <0.001 | -0.65 (0.07), <0.001 | -0.49 (0.10), <0.001 |
| Marital status |  |  |  |  |
| Unmarried vs. others | 2.01 (1.58 – 2.55), <0.001 | 1.17 (1.01 – 1.36), 0.041 | 0.23 (0.33), 0.48 | 0.1 (0.46), 0.831 |
| Married/partnership vs. others | 0.95 (0.84 – 1.09), 0.494 | 0.99 (0.91 – 1.09), 0.896 | 0.28 (0.22), 0.189 | 0.54 (0.30), 0.074 |
| **Random effect variance** |  |  |  |  |
| Period | 0.027 | 0.004 | 0.441 | 0 |
| ICC | 0.01* | <0.01* | 0.02* | NA |
| **Model fitness** |  |  |  |  |
| Bayesian information criterion | 17739.9 | 30642.8 | 42935.6 | 20171.4 |
| Marginal *R*^2^ | 0.058 | 0.020 | 0.049 | 0.033 |
| Conditional *R*^2^ | 0.065 | 0.022 | 0.066 | NA |
| †Adjusted for sex, ethnicity, (residence), education, and marital status; ‡Age and education were centered around grand means.  *ICC is significant with *p*-value <0.05; ** with *p*-value <0.01. OR: odds ratio; CI: confidence interval; SE: standard error; ICC: intraclass correlation coefficient. | | | | |

| **Table S4**. Results for the linear regression model of traditional filial piety score in urban residents (n = 3,339) | |
| --- | --- |
| **Predictors** | **Estimate (SE), *p*-value** |
| Intercept | 38.23 (1.4), <0.001 |
| Age† | -0.12 (0.55), 0.830 |
| Age^2^ | 0.07 (0.05), 0.218 |
| Period (2017 vs. 2006) | -0.6 (0.63), 0.341 |
| Cohort |  |
| 1940-4 vs. before 1939 | 0.05 (0.62), 0.933 |
| 1945-9 vs. before 1939 | -0.44 (0.77), 0.569 |
| 1950-4 vs. before 1939 | 0 (0.99), 0.997 |
| 1955-9 vs. before 1939 | 0.18 (1.22), 0.885 |
| 1960-4 vs. before 1939 | -0.44 (1.48), 0.767 |
| 1965-9 vs. before 1939 | -0.22 (1.73), 0.899 |
| 1970-4 vs. before 1939 | -0.2 (1.97), 0.917 |
| 1975-9 vs. before 1939 | -0.81 (2.26), 0.721 |
| 1980-4 vs. before 1939 | -0.86 (2.52), 0.734 |
| 1985-9 vs. before 1939 | -1.26 (2.78), 0.651 |
| After 1990 vs. before 1939 | -1.33 (3.18), 0.675 |
| Sex (female vs. male) | 0.47 (0.17), 0.006 |
| Ethnicity (Han vs. non-Han) | -1.32 (0.41), 0.001 |
| Education† | -0.48 (0.1), <0.001 |
| Marital status |  |
| Unmarried vs. others | 0.11 (0.46), 0.82 |
| Married/partnership vs. others | 0.55 (0.31), 0.07 |
| **Model fitness** |  |
| Bayesian information criterion | 20152.14 |
| *R*^2^ | 0.033 |
| Adjusted *R*^2^ | 0.028 |
| †Age and education were centered around grand means. SE: standard error. | |

| **Table S5**. Results for the crude hierarchical age-period-cohort model of old-age pension coverage, elder-care responsibility conception, and traditional filial piety score | | | | | | |
| --- | --- | --- | --- | --- | --- | --- |
| **HAPC model** | **Old-age pension coverage (n = 55,541)** | **Elder-care primary responsibility (n = 56,332)** | | | | **Filial piety score (n = 7,085)** |
|  |  | **Government** | **Offspring** | **Self** | **Sharing** |  |
| **Fixed effect** | **OR (95% CI), *p*-value** | **OR (95% CI), *p*-value** | **OR (95% CI), *p*-value** | **OR (95% CI), *p*-value** | **OR (95% CI), *p*-value** | **Estimate (SE), *p*-value** |
| Intercept | 2.05 (1.36 – 3.10), 0.001 | 0.11 (0.10 – 0.13), <0.001 | 0.99 (0.86 – 1.14), 0.924 | 0.06 (0.05 – 0.06), <0.001 | 0.52 (0.47 – 0.58), <0.001 | 37.81 (0.10), 0.001 |
| Age† | 1.3 (1.23 – 1.37), <0.001 | 1.3 (1.26 – 1.35), <0.001 | 0.9 (0.86 – 0.93), <0.001 | 1.52 (1.45 – 1.59), <0.001 | 0.96 (0.93 – 0.99), 0.005 | 0.38 (0.040), <0.001 |
| Age^2^ | 0.96 (0.94 – 0.98), <0.001 | 0.97 (0.95 – 0.99), <0.001 | 1.02 (1.01 – 1.04), <0.001 | 0.94 (0.92 – 0.96), <0.001 | 0.98 (0.96 – 1.00), 0.043 | -0.06 (0.02), 0.004 |
| **Random effects variance** |  |  |  |  |  |  |
| Cohort | 0.036 | 0.007 | 0.016 | 0.008 | 0.008 | 0.000 |
| Period | 0.204 | 0.022 | 0.015 | 0.017 | 0.007 | 0.007 |
| ICC | 0.07* | 0.01* | 0.01* | 0.01* | 0.001 | NA |
| **Model fitness** |  |  |  |  |  |  |
| Bayesian information criterion | 67187.7 | 35830.6 | 77173.7 | 22721.9 | 71352.3 | 43077.6 |
| Marginal *R*^2^ | 0.047 | 0.052 | 0.009 | 0.120 | 0.002 | 0.013 |
| Conditional *R*^2^ | 0.112 | 0.060 | 0.019 | 0.127 | 0.007 | NA |
| †Age was centered around grand means.  *ICC is significant with *p*-value <0.05; ** with *p*-value <0.01. OR: odds ratio; CI: confidence interval; SE: standard error; ICC: intraclass correlation coefficient. | | | | | | |

| **Table S6**. The marginal effects of the age-period-cohort analysis by residence using the generalized additive model | | | | | | | | | | | | | | | |
| --- | --- | --- | --- | --- | --- | --- | --- | --- | --- | --- | --- | --- | --- | --- | --- |
| **Effect†** | **Old-age pension coverage (n = 55,541)** | | | **Elder-care primary responsibility (n = 56,332)** | | | | | | | | | | | |
|  |  |  |  | **Government** | | | **Offspring** | | | **Self** | | | **Sharing** | | |
|  | **Age, *y*** | **Period** | **Cohort** | **Age, *y*** | **Period** | **Cohort** | **Age, *y*** | **Period** | **Cohort** | **Age, *y*** | **Period** | **Cohort** | **Age, *y*** | **Period** | **Cohort** |
| **In total** | | | | | | | | | | | | | | | |
| Value with maximum OR | 72 | 2017 | 1942 | 76 | 2017 | 1937 | 20 | 2010 | 1990 | 75 | 2016 | 1937 | 69 | 2014 | 1945 |
| Maximum OR | 1.55 | 1.43 | 1.53 | 1.66 | 1.25 | 1.65 | 1.92 | 1.34 | 1.8 | 2.1 | 1.19 | 2.12 | 1.17 | 1.12 | 1.17 |
| Value with minimum OR | 20 | 2010 | 1997 | 20 | 2010 | 1995 | 71 | 2015 | 1942 | 20 | 2010 | 1997 | 90 | 2010 | 1920 |
| Minimum OR | 0.25 | 0.42 | 0.3 | 0.31 | 0.76 | 0.29 | 0.61 | 0.9 | 0.61 | 0.25 | 0.8 | 0.24 | 0.71 | 0.86 | 0.61 |
| Ratio (Maximum/Minimum OR) | 6.18 | 3.38 | 5.08 | 5.44 | 1.64 | 5.72 | 3.14 | 1.48 | 2.95 | 8.28 | 1.49 | 8.68 | 1.64 | 1.29 | 1.93 |
| *R*^2^ | 0.161 | | | 0.031 | | | 0.075 | | | 0.029 | | | 0.032 | | |
| **In urban residents** | | | | | | | | | | | | | | | |
| Value with maximum OR | 73 | 2017 | 1942 | 75 | 2017 | 1939 | 20 | 2010 | 1990 | 75 | 2016 | 1937 | 55 | 2015 | 1957 |
| Maximum OR | 1.62 | 1.43 | 1.62 | 1.69 | 1.25 | 1.67 | 2.42 | 1.31 | 2.27 | 2.66 | 1.24 | 2.71 | 1.08 | 1.11 | 1.08 |
| Value with minimum OR | 20 | 2010 | 1997 | 20 | 2010 | 1995 | 72 | 2016 | 1942 | 25 | 2010 | 1990 | 20 | 2017 | 1920 |
| Minimum OR | 0.17 | 0.59 | 0.18 | 0.34 | 0.8 | 0.33 | 0.49 | 0.88 | 0.5 | 0.29 | 0.8 | 0.29 | 0.87 | 0.9 | 0.81 |
| Ratio (Maximum/Minimum OR) | 9.39 | 2.4 | 8.75 | 5.03 | 1.56 | 5.04 | 4.9 | 1.48 | 4.55 | 9.12 | 1.54 | 9.33 | 1.24 | 1.22 | 1.32 |
| *R*^2^ | 0.139 | | | 0.028 | | | 0.046 | | | 0.042 | | | 0.017 | | |
| **In rural residents** | | | | | | | | | | | | | | | |
| Value with maximum OR | 69 | 2017 | 1945 | 90 | 2017 | 1927 | 20 | 2010 | 1920 | 64 | 2017 | 1949 | 71 | 2013 | 1943 |
| Maximum OR | 1.49 | 1.57 | 1.48 | 1.67 | 1.25 | 1.68 | 1.71 | 1.39 | 1.83 | 1.71 | 1.15 | 1.74 | 1.24 | 1.18 | 1.24 |
| Value with minimum OR | 20 | 2010 | 1920 | 20 | 2010 | 1995 | 71 | 2014 | 1943 | 20 | 2010 | 1997 | 90 | 2010 | 1920 |
| Minimum OR | 0.29 | 0.25 | 0.27 | 0.29 | 0.73 | 0.26 | 0.68 | 0.87 | 0.68 | 0.27 | 0.79 | 0.2 | 0.73 | 0.79 | 0.49 |
| Ratio (Maximum/Minimum OR) | 5.06 | 6.17 | 5.39 | 5.85 | 1.73 | 6.44 | 2.5 | 1.6 | 2.69 | 6.36 | 1.46 | 8.59 | 1.69 | 1.49 | 2.54 |
| *R*^2^ | 0.144 | | | 0.015 | | | 0.021 | | | 0.009 | | | 0.014 | | |
| †Adjusted for sex, ethnicity, (residence), education, and marital status; OR: odds ratio. | | | | | | | | | | | | | | | |

| **Table S7**. Results for the hierarchical age-period-cohort model of traditional filial piety score after removing questions related to patrilineality and gender norms by residence | | | |
| --- | --- | --- | --- |
| **HAPC model†** | **Total (n = 7,085)** | **Urban (n = 3,339)** | **Rural (n = 3,746)** |
| **Mean filial piety score, mean ± SD** | 27.92 ± 3.68 | 27.63 ± 3.65 | 28.19 ± 3.70 |
| **Fixed effect, (Estimate (SE), *p*-value)** |  |  |  |
| Intercept | 28.25 (0.27), <0.001 | 28.14 (0.42), <0.001 | 28.45 (0.54), <0.001 |
| Age‡ | 0.06 (0.04), 0.104 | 0.02 (0.06), 0.797 | 0.09 (0.05), 0.131 |
| Age^2^ | -0.04 (0.02), 0.049 | 0 (0.03), 0.877 | -0.06 (0.03), 0.042 |
| Sex (female vs. male) | 0 (0.09), 0.970 | 0.12 (0.13), 0.365 | -0.1 (0.12), 0.417 |
| Ethnicity (Han vs. non-Han) | -0.53 (0.18), 0.003 | -0.63 (0.31), 0.041 | -0.45 (0.22), 0.042 |
| Registered residence (rural vs. region) | 0.25 (0.1), 0.016 |  |  |
| Education‡ | -0.24 (0.05), <0.001 | -0.18 (0.07), 0.012 | -0.28 (0.08), <0.001 |
| Marital status |  |  |  |
| Unmarried vs. others | 0.05 (0.24), 0.835 | -0.21 (0.33), 0.531 | 0.26 (0.34), 0.452 |
| Married/partnership vs. others | 0.12 (0.16), 0.465 | 0.18 (0.23), 0.419 | 0.03 (0.22), 0.888 |
| **Random effects variance** |  |  |  |
| Cohort | 0.000 | 0.005 | 0.003 |
| Period | 0.030 | 0.072 | 0.389 |
| ICC | NA | 0.006 | 0.028* |
| **Model fitness** |  |  |  |
| Bayesian information criterion | 38622.5 | 18195.8 | 20462.5 |
| Marginal *R*^2^ | 0.013 | 0.007 | 0.010 |
| Conditional *R*^2^ | NA | 0.012 | 0.038 |
| †Adjusted for sex, ethnicity, (residence), education, and marital status; ‡Age and education were centered around grand means.  *ICC is significant with *p*-value <0.05; ** with *p*-value <0.01. SD: standard deviation; SE: standard error; ICC: intraclass correlation coefficient | | | |
